# Supplementary material for: Complexome profiling of the Chlamydomonas psb28 mutant reveals TEF5 as an early PSII assembly factor
Source: Plant Cell. 2025 Mar 17;37(6):koaf055. doi: 10.1093/plcell/koaf055 (PMC12164590; doi:10.1093/plcell/koaf055)
Supplement: koaf055_Supplementary_Data [file koaf055_supplementary_data.zip › psb28_interactive.zip!/plots/intensity/qualityPlots/peptides/_Cre13.g569750.t1.2.html]

### Cre13.g569750.t1.2

Trivial name:   
SeqCoverage [median]: 26.70  
Mass: 19.94 kDa  
  
 

| Sequence | MissedCleavage | Mass [Da] | wtIntensity | muIntensity | Score |
| --- | --- | --- | --- | --- | --- |
| AEAPLEDR | 0 | 899.43 | 3902.48 | 17605.57 | 125.31 |
| TGETTELGQPK | 0 | 1159.57 | 242.17 | 1478.75 | 169.41 |
| TGETTELGQPKPGGR | 1 | 1526.77 | 18204.47 | 30420.88 | 136.99 |
| TPAASPDADGDKQPVATAPATVPESASSSPATR | 1 | 3149.51 | 0.00 | 1717.03 | 35.13 |
|  |  |  | 22349.1 | 51222.2 |  |
